# Reconstruction of mandibular defects in osteoradionecrosis and medication-related osteonecrosis of the jaw using fibula free flap and management of postoperative wound infections

**DOI:** 10.1186/s40902-022-00366-2

**Published:** 2022-12-09

**Authors:** Hyemin Oh, Dohyun Kwon, Jaemyung Ahn, Jun-Young Paeng

**Affiliations:** Department of Oral and Maxillofacial Surgery, Samsung Medical Center, Sungkyunkwan University School of Medicine, 81, Irwon-ro, Gangnam-gu, Seoul, 06351 Republic of Korea

**Keywords:** Osteoradionecrosis (ORN), Medication-related osteonecrosis of the jaws (MRONJ), Mandible, Fibula free flap, Infection

## Abstract

**Background:**

Complications from osteoradionecrosis (ORN) and medication-related osteonecrosis of the jaw (MRONJ) include oro-cutaneous fistulas, necrotic bone exposure, soft-tissue defects, and pathologic fractures. The fibula free flap (FFF) is a common free flap method used to reconstruct the mandible in severe cases. Recently, we have used the FFF successfully for the reconstruction of ORN and MRONJ mandibular defects. We report this method as a recommended technique for the treatment of ORN and MRONJ and the management method of postoperative infections.

**Methods:**

Four patients who were diagnosed with ORN of the mandible and 3 patients who were diagnosed with MRONJ of the mandible were included in the study. Among the 7 patients, 3 patients also had pathologic fractures. Partial mandibulectomy and FFF reconstruction were performed at the Department of Oral and Maxillofacial Surgery, Samsung Medical Center from April 2019 to March 2021.

**Results:**

All 7 patients recovered following the reconstruction of the defect by FFF. Four patients experienced infections after surgery and pus cultures were performed. All were well healed without flap damage after changing the antibiotics by consultation with infectious medicine experts.

**Conclusion:**

FFF is a widely used method and can provide an extensive flap to reconstruct the mandible, especially those affected by ORN or MRONJ. If an infection occurs after surgery, appropriate antibiotic changes should be made through cooperation with the infectious medicine department. Therefore, FFF is a well-established and recommended method even in cases of challenging reconstruction.

## Background

Osteoradionecrosis (ORN) is a severe complication after radiotherapy (RT) for head and neck tumors where the radiated bone becomes necrotic and exposed. It can be defined as a condition in which the irradiated bone becomes exposed through a wound in the overlying mucosa or skin with a fistula [1]. Medication-related osteonecrosis of the jaw (MRONJ) is defined as non-healing, exposed necrotic bone or bone that can be probed through a fistula in the maxillofacial area for at least 8 weeks in a patient with a history of anti-resorptive or anti-angiogenic agent use in the absence of radiation exposure to the head and neck region [2]. The main risk factors of ORN and MRONJ are dental extractions and trauma to the bone [3]. The symptoms are oro-cutaneous fistulas, necrotic bone exposure, soft tissue defects, and pathologic fractures. Decortication of superficial bone structures, the removal of small sequestra, and sequestrectomy are used to treat ORN and MRONJ. If those treatments fail, mandibulectomy is chosen as the last method, and reconstruction is also performed to rehabilitate the wound. Reconstruction of ORN defects is usually more difficult because of radiation damage, fibrosis of the defect, and the complex defect environment [4]. The most commonly used flap for reconstruction of the defective area after mandibulectomy is fibula free flap (FFF). It provides sufficient bone length and bone properties similar to the mandible, so it is suitable for the installation of dental implants after reconstruction and provides aesthetic and functional recovery [5, 6]. It also helps to cover soft tissue areas because it can also get the skin layer. In addition, a two-team approach is available during surgery to remove the necrotic lesion and harvest FFF simultaneously. Recently, we resected ORN and MRONJ areas and reconstructed them using FFF and managed postoperative infections using appropriate antibiotics with consultation with the infectious medicine department and report good results.

## Patients and methods

### Patients

Four patients who were diagnosed with ORN of the mandible and 3 patients who were diagnosed with MRONJ of the mandible were included in the study. One of them was diagnosed simultaneously with ORN and MRONJ. Among the 7 patients, 3 patients also had pathologic fractures. All patients underwent surgery with wide dissection of the oral cavity and partial mandibulectomy. The defects were reconstructed with the FFF method at Samsung Medical Center from April 2019 to March 2021. This study was approved by Institutional Review Board of our hospital.

**Fig. 1:**
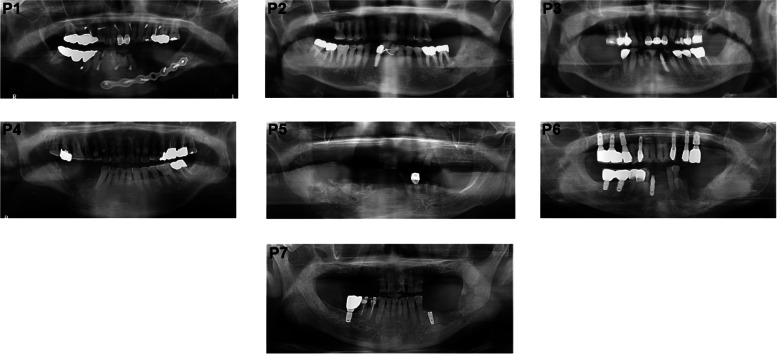
Preoperative orthopantomogram X-rays of the patients

All patients had mandibular defect (Fig. 1). The data of the 7 patients and whether they had radiation therapy, the drugs they took, the presence of a fistula before surgery, and the presence of a pathological fracture are listed (Table 1). Patient 1 was diagnosed with adenoid cystic carcinoma of the mouth floor and tongue, then underwent near total glossectomy and left anterolateral thigh (ALT) flap at another hospital (ENT) 3 years and 8 months earlier. Patient 7 underwent mandibulectomy due to squamous cell carcinoma (SCC) of the left retromolar area at another hospital (in the Department of Oral and Maxillofacial Surgery) 2 years and 9 months earlier. Patients 1, 2, 3, 4, and 7 received radiation after their cancer diagnosis and patients 1 and 7 did not have medical record information regarding the total radiation dose. Patients 3, 5, and 6 were diagnosed with MRONJ. Patients 1, 5, 6, and 7 had a fistula before surgery. Patients 1, 4, and 7 had pathologic fractures of the mandible.

**Table 1:** Summary of preoperative patient data

	P1	P2	P3	P4	P5	P6	P7
**Age**	65	69	73	77	80	75	70
**Sex**	F	M	M	M	F	F	M
**Last surgery**	3Y 8MA: Near total glossectomy and Lt. ALT flap	X	X	X	X	X	2Y 9MA: Resection of Lt. Mn. Retromolar area due to SCC
**RT**	N/A / 33Fxs.	68.4Gy/30Fxs.	68.4Gy/30Fxs.	66Gy/33Fxs.	X	X	N/A
**Medication history of anti-resorptive agent or anti-angiogenic agent**	X	X	**Multiple lung meta** Cetuximab & cisplatin for 7W Monotaxel for 13W Nivolumab 1 time Weekly MTX 8 times	X	Osteoporosis Mx.	Osteoporosis Mx. PO for 2Y Osteoporosis Mx. Injection for 2~3Y	X
**Fistula**	O	X	N/A	X	O	O	O
**Pathologic fracture**	O	X	X	O	X	X	O

*Abbreviations*: *RT* radiation treatment, *Lt. ALT* left anterolateral thigh, *meta* metastasis, *Mx* medication, *PO* peroral, *SCC* squamous cell carcinoma

## Methods

All surgeries were performed by the same 2 operators (JY.P, JM.A.). Before surgery, surgical stents, and guides were made using a three-dimensional (3D) simulation, and the accuracy was checked according to the rapid prototyping (RP) model. If a patient was admitted to the hospital, the surgeon would mark the perforators on the patient’s leg. The operation was performed under general anesthesia. One surgeon dissected the oral cavity and removed the necrotic bone. The other surgeon designed the flap on the leg and elevated the flap using a surgical stent. The guide was aligned with the bone of the FFF, then the bone was shaped and fixed with metal plates and screws. The defect was reconstructed with an FFF and vessels were anastomosed. Primary suturing was done at the donor site without other grafts, and Dermabond was applied to the incision line. After surgery, the patient was sent to the intensive care unit for a day for respiratory care and moved to the general ward after ventilator weaning. During the hospitalization period, follow-up observations were performed through daily laboratory tests and dressing checks. In particular, the surgical area, including the flap, was carefully observed for the development of complications. Of the 7 patients, 4 had complications, which were infections accompanied by increases in C-reactive protein (CRP) levels. Three patients underwent surgery due to ORN, and one had ORN accompanied by MRONJ. After obtaining the pus culture results, we consulted with the infectious medicine department and the antibiotics of all patients were changed according to the results.

## Results

**Fig. 2:**
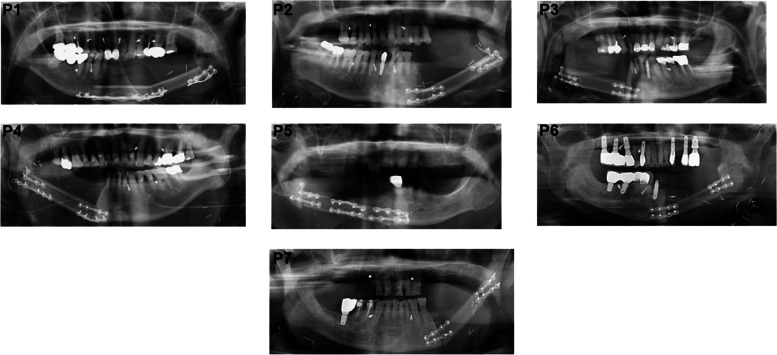
Postoperative orthopantomogram X-rays of the patients

All of the FFF reconstructions were successful (Fig. 2). Flap survival, follow-up periods, postoperative infections, fistulas, and bone necrosis are listed (Table 2). Postoperative infections were observed in 4 patients and fistulas in 4 patients. No patients showed bone necrosis. The FFFs were well sustained in all patients.

**Table 2:** Summary of postoperative patient data

	P1	P2	P3	P4	P5	P6	P7
**Age**	65	69	73	77	80	75	70
**Sex**	F	M	M	M	F	F	M
**RT**	N/A/33Fxs.	68.4Gy/30Fxs.	68.4Gy/30Fxs.	66Gy/33Fxs.	x	x	N/A
**Medication history of anti-resorptive agent or anti-angiogenic agent**	X	X	**Multiple lung meta** Cetuximab & Cisplatin for 7W Monotaxel for 13W Nivolumab 1 time Weekly MTX 8 times	X	Osteoporosis Mx.	Osteoporosis Mx. PO for 2Y Osteoporosis Mx. Injection for 2~3Y	X
**Fistula**	O	X	N/A	X	O	O	O
**Pathologic Fracture**	O	X	X	O	X	X	O
**Flap Survival**	O	O	O	O	O	O	O
**F/U period**	2M	6M	8M	6M	4M	1M	5W
**Postoperative infection**	O	O	O	X	X	X	O
**Postoperative fistula**	O	X	O	X	X	X	O
**Postoperative bone necrosis**	X	X	X	X	X	X	X

*Abbreviations*: *RT* radiation treatment, *F/U* follow-up, *meta* metastasis, *Mx* medication, *PO* peroral

Four patients experienced infections after surgery. The average time of infection was 9.5 days after surgery. The pus culture results of the 4 patients (patients 1, 2, 3, and 7) with postoperative infections are listed (Table 3). *Enterococcus faecalis* was identified in patient 1 and was susceptible to penicillin (penicillin-G, ampicillin), carbapenem (imipenem), aminoglycoside (streptomycin), quinolone (ciprofloxacin, levofloxacin, and norfloxacin), linezolid, teicoplanin, vancomycin, tigecycline, and nitrofurantoin. *Acinetobacter* was identified in patient 2 and was susceptible to penicillin (ampicillin/sulbactam, ticarcillin/clavulanic acid, and piperacillin/tazobactam), carbapenem (imipenem and meropenem), aminoglycoside (gentamicin), tetracyline (minocycline and tigecycline), and trimethoprim/sulfamethoxazole. *Streptococcus mitis* and *streptococcus oralis* were identified in patient 3 and were susceptible to cephalosporin (cefotaxime and ceftriaxone), quinolone (levofloxacin and moxifloxacin), a macrolide (erythromycin), lincomycin (clindamycin), linezolid, vancomycin, tetracycline (tigecyline), and chloramphenicol. *Pseudomonas aeruginosa* was identified in patient 7 and was susceptible to penicillin (ticarcillin/clavulanic acid, piperacillin, and piperacillin/tazobactam), cephalosporin (ceftazidime and cefepime), aztreonam, carbapenem (imipenem and meropenem), aminoglycoside (amikacin and gentamicin), and quinolone (ciprofloxacin). After receiving the pus culture results, we consulted with the infectious medicine department and the antibiotics of all patients were changed according to the results. The antibiotics of patient 1 were changed 4 days after infection, 2 days after infection in patient 2, and immediately on the day of infection in patient 3. On average, the antibiotics were changed 2 days after infection. The initial antibiotics used were ceftriaxone and flomoxef, which were changed to tazoferan (piperacillin/tazobactam), vancomycin, and ampicillin/sulbactam.

**Table 3:** Pus culture results

	Antibiotics	MIC	Susceptibility
P1	Penicillin-G	4	S
	Ampicillin	≤2	S
	Ampicillin/sulbactam	≤2	S
	Imipenem	≤1	S
	Gentamicin high level	SYN-R	R
	Streptomycin high-level resistance	SYN-S	S
	Ciprofloxacin	≤ 0.5	S
	Levofloxacin	0.5	S
	Norfloxacin	2	S
	Erythromycin	≥ 8	R
	Clindamycin	≥ 8	R
	Quinupristin/dalfopristin	4	R
	Linezolid	2	S
	Teicoplanin	≤ 0.5	S
	Vancomycin	1	S
	Tetracycline	≥ 16	R
	Tigecycline	≤ 0.12	S
	Nitrofurantoin	≤ 16	S
	Trimethoprim/sulfamethoxazole	≤ 10	R
**P2**	Ampicillin/sulbactam	≤ 2	S
	Ticarcillin/clavulanic acid	≤ 8	S
	Piperacillin	32	I
	Piperacillin/tazobactam	≤ 4	S
	Cefotaxime	32	I
	Ceftazidime	16	I
	Cefepime	16	I
	Aztreonam	32	R
	Imipenem	≤ 0.25	S
	Meropenem	≤ 0.25	S
	Gentamicin	≤ 1	S
	Ciprofloxacin	≥ 4	R
	Minocycline	≤ 1	S
	Tigecycline	≤ 0.5	S
	Trimethoprim/sulfamethoxazole	≤ 20	S
**P3**	Penicillin-G	1	I
	Ampicillin	4	I
	Cefotaxime	0.5	S
	Ceftriaxone	0.5	S
	Levofloxacin	1	S
	Moxifloxacin	0.12	S
	Erythromycin	≤ 0.12	S
	Clindamycin	≤ 0.25	S
	Linezolid	≤ 2	S
	Vancomycin	0.5	S
	Tetracycline	2	S
	Tigecycline	≤ 0.06	S
	Chloramphenicol	2	S
**P7**	Ampicillin/sulbactam	≥ 32	R
	Ticarcillin/clavulanic acid	16	S
	Piperacillin	≤ 4	S
	Piperacillin/tazobactam	8	S
	Cefotaxime	16	R
	Ceftazidime	4	S
	Cefepime	2	S
	Aztreonam	2	S
	Imipenem	1	S
	Meropenem	≤ 0.25	S
	Amikacin	≤ 2	S
	Gentamicin	4	S
	Ciprofloxacin	≤ 0.25	S
	Minocycline	≥ 16	R
	Tigecycline	≥ 8	R
	Trimethoprim/sulfamethoxazole	160	R

The history of other treatments and surgeries before surgery in this hospital, the date of surgery in this hospital, the time of infection, the presence or absence of incision and drainage (I&D), and antibiotic changes are listed (Table 4).

**Table 4:** Summary of patients with postoperative infections

	P1	P2	P3	P7
**Pre Op. treatment**	2016.08 Near total glossectomy, left MRND, left ALT flap reconstruction due to tongue cancer	2019.05.23 Left maxilla sequestrectomy	2019.08.21 Sequestrectomy and saucerization on #44–47 and extraction of #47	2018.06.19 Resection of left mandible retromolar area, both selective neck dissection due to SCC
**Op. date**	2019.04	2019.09	2019.12	2021.03
**Onset**	POD 6	POD 14	POD 10	POD 8
**I&D**	O	X	O	O
**Culture**	O	O	O	O
**Antibiotics**	2019.04.13–2019.04.24 Ceftriaxone sod 2 g IV	2019.09.16–2019.10.01 Flomoxef 1000 mg bid IV	2019.12.02–2019.12.12 Flomoxef 1000 mg bid IV	2021.03.14–2021.03.26 Flomoxef 1000 mg bid IV
	2019.04.25 Tigecylibe 50 mg bid IV	2019.10.02–2019. 10.14 Ampicillin & sulbactam 3 g qid IV	2019.12.13–1019.12.15 Tazoferan 4.5 g tid INF Vancomycin 1 g bid IV	2021.03.27–2021.04.02 Tazoferan 4.5 g qid INF
	2019.04.26–2019. 04.29 Tazoferan 4.5 g tid INF	2019.10.15–2019. 10. 22 Amoxicillin & clavulanic acid 375 mg tid	2019.12.16–2019.12.30 Ampicillin & sulbactam 3 g qid IV	
	2019.04.30–2019.05.14 Ampicillin & sulbactam 3.0 g qid IV		2019.12.31–2020.01.09 Cefixime 200 mg bid	

*Tazoferan* piperacillin/tazobactam, *Flomoxef* third-generation cephalosporin

*Abbreviations*: *Pre-op.* preoperative, *Op*. operation, *I & D* incision and drainage, *MRND* modified radical neck dissection, *ALT* anterolateral thigh, *POD* postoperative date, *IV* intravenous, *bid* bis in die, *tid* ter in die, *qid* quarter in die, *SCC* squamous cell carcinoma

**Fig. 3:**
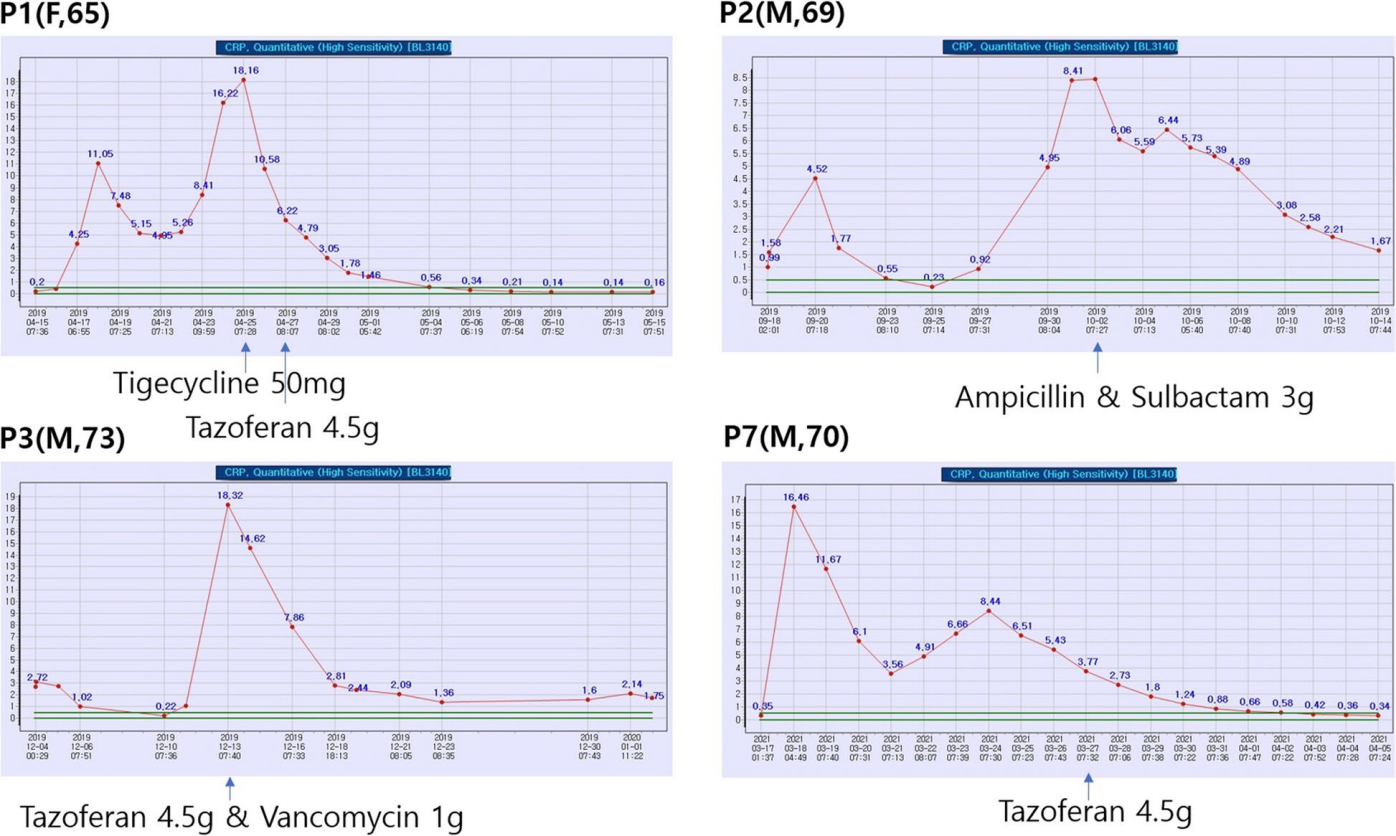
Changes in CRP levels with antibiotic changes

The CRP levels were changed according to antibiotic changes (Fig. 3). A decrease in CRP levels was observed in all patients after proper antibiotic changes. The 4 patients with postoperative infections were cured by antibiotic changes without affecting the flap. When patients receive antibiotics for a long time due to chronic infections, infections caused by antibiotic-resistant bacteria after surgery are likely to occur. Therefore, if antibiotics are changed and used for an appropriate time by cooperation with the infectious medicine department, the flap can be well-maintained and healing can be promoted.

## Discussion

The treatment methods for ORN and MRONJ include conservative approaches and surgical intervention. Treatment should be approached in steps according to the stage of necrotic progression. Conservative treatment is generally performed in early-stage ORN and MRONJ. Antibiotics, debridement, hyperbaric oxygen therapy, and pharmacotherapy are representative methods of conservative treatment [7–9]. However, if conservative treatment does not work for a long time, a surgical approach should be attempted immediately regardless of the stage. A surgical approach is essential for advanced ORN or MRONJ accompanied by fractures, osteomyelitis, oro-cutaneous fistula, or intractable pain. Radical debridement, mandibulectomy, and free flap reconstruction are commonly used as surgical methods [10–13]. The most commonly used flap for the reconstruction of the defective area after mandibulectomy is a FFF. The pedicle length of the FFF is sufficient to reach the transverse cervical vessels in the case of using the distal bone and removing the proximal bone [4]. The mandibular defects in our patients severely damaged with ORN or MRONJ were successfully reconstructed using FFFs.

Common complications after successful reconstruction include flap loss, fistula, neck infections, and hematomas, which require additional surgery. Minor complications that do not require surgery include donor site dehiscence, infections, and partial skin graft loss [4]. All of our patients with postoperative infections were ORN patients (patient 3 also had MRONJ). According to previous studies, the incidence of complications after the reconstruction of ORN defects with a free flap ranged from 24 to 44% [14–17]. Of the complications after ORN defect reconstruction, 13% were due to infections [15, 18, 19]. In our study, 4 of 5 patients with ORN developed infections with elevated CRP levels after surgery. However, this should be regarded as a limitation of this study due to the relatively insufficient number of patients, and it should be supplemented with studies of larger patient groups in the future.

Alam et al. reported that 6 of 33 ORN patients (18%) had wound infections as postoperative complications. Four of 6 patients with these postoperative infections did not show the growth of typical polymicrobial anaerobic oral flora in the cultures but instead grew single-organism multi-resistant gram-negative rods. The organisms were resistant to penicillin and clindamycin, the 2 typical antibiotics of choice for orally contaminated wounds. However, they did not mention what antibiotics they changed to and how they controlled the infections [17].

In 2021, Zhu et al. reported that 173 (79.0%) of 219 samples from the surface of local infected lesions or exudate liquid showed significant bacterial infections. The top 3 aerobic bacteria were *Klebsiella pneumoniae* (15.1%), *Pseudomonas aeruginosa* (13.54%), and *Staphylococcus aureus* (10.94%). Methicillin-resistant *Staphylococcus aureus* (MRSA) accounted for 5.21% in the whole samples. The authors reported the antimicrobial susceptibilities of all culture-positive strains and the drug resistance rate (DRR). The drugs with almost no resistance were ticarcillin (DRR = 0.00%), ofloxacin (DRR = 0.00%), vancomycin (DRR = 0.00%), tigecycline (DRR = 0.00%), meropenem (DRR = 0.88%), and piperacillin + tazobactam (DRR = 0.88%) [20].

Gram-negative bacteria (*Acinetobacter nosocomialis* and *Pseudomonas aeruginosa*) and gram-positive bacteria (*Enterococcus faecalis*, *Streptococcus mitis*, and *Streptococcus oralis*) were detected in our patients’ pus cultures. After surgery, ceftriaxone and flomoxef were prophylactically used, and after the infection occurred, it was changed to tazoferan (piperacillin/tazobactam), vancomycin, ampicillin/sulbactam, or other antibiotics by consultation with the infectious medicine department regarding the pus culture results. Antibiotics were changed immediately or the next day according to the advice from infectious medicine. The time taken until CRP levels were reduced to less than 2mg/dL was 6 days for patient 1, 11 days for patient 2, 10 days for patient 3, and 11 days for patient 7. On average, CRP levels were controlled 9.5 days after infection and all patients who developed infections were discharged after the infection was controlled.

Empirical antibiotic use is also important, but to use antibiotics suitable for each patient, it is important to cultivate the pus or blood from the infection focus and consult with infectious medicine experts to select the appropriate antibiotics. If complication management after surgery can be thoroughly performed, FFF is a very useful method for reconstructing ORN and MRONJ defect sites.

This study is limited because it has small number of cases (7 patients). Reconstruction of mandibular defect in ORN and MRONJ using FFF is not common currently. Hence, more cases should be collected to evaluate prognosis of FFF and management of postoperative wound infections in the future.

## Conclusion

The fibula free flap is a widely used method and can provide an extensive flap to reconstruct the mandible, especially when it is affected by ORN or MRONJ. In addition, if an infection occurs after surgery, appropriate antibiotic changes should be made through cooperation with the infectious medicine department. Therefore, the fibula free flap is a well-established and recommended method, even in cases of challenging reconstruction.

## Data Availability

The datasets used and/or analyzed during the current study are available from the corresponding author on reasonable request.

## Abbreviations

ORN: Osteoradionecrosis
MRONJ: Medication-related osteonecrosis of the jaw
FFF: Fibula free flap
RT: Radiotherapy
ALT: Anterolateral thigh
ENT: Ear, nose, and throat
SCC: Squamous cell carcinoma
3D: Three-dimensional
RP: Rapid prototyping
CRP: C-reactive protein
MRSA: Methicillin-resistant *Staphylococcus aureus*
DRR: Drug resistance rate
